# Neuroinflammation at the Gray–White Matter Interface in Active-Duty U.S. Special Operations Forces

**DOI:** 10.1089/neur.2024.0116

**Published:** 2024-12-16

**Authors:** Brian L. Edlow, Chieh-En J. Tseng, Natalie Gilmore, Isabella R. McKinney, Samantha L. Tromly, Katryna B. Deary, Collin G. Hu, Brian C. Healy, David S. Priemer, Christine L. Mac Donald, Kristen Dams-O’Connor, Douglas N. Greve, Yelena G. Bodien, Daniel P. Perl, Jacob M. Hooker, Nicole R. Zürcher

**Affiliations:** ^1^ Department of Neurology, Center for Neurotechnology and Neurorecovery, Massachusetts General Hospital and Harvard Medical School, Boston, Massachusetts, USA.; ^2^ Department of Radiology, Athinoula A. Martinos Center for Biomedical Imaging, Massachusetts General Hospital and Harvard Medical School, Boston, Massachusetts, USA.; ^3^ Institute for Applied Engineering, University of South Florida, Tampa, Florida, USA.; ^4^ United States Special Operations Command, MacDill Air Force Base, Tampa, Florida, USA.; ^5^ Navy SEAL Foundation, Virginia Beach, Virginia, USA.; ^6^ Department of Family Medicine, F. Edward Hébert School of Medicine, Uniformed Services University of the Health Sciences, Bethesda, Maryland, USA.; ^7^ Biostatistics Center, Massachusetts General Hospital, Boston, Massachusetts, USA.; ^8^ Department of Neurology, Brigham and Women’s Hospital, Boston, Massachusetts, USA.; ^9^ Department of Pathology, F. Edward Hébert School of Medicine, Uniformed Services University, Bethesda, Maryland, USA.; ^10^ Department of Neurological Surgery, University of Washington, Seattle, Washington, USA.; ^11^ Department of Rehabilitation and Human Performance, Icahn School of Medicine at Mount Sinai, New York, New York, USA.; ^12^ Department of Neurology, Icahn School of Medicine at Mount Sinai, New York, New York, USA.; ^13^ Department of Physical Medicine and Rehabilitation, Spaulding Rehabilitation Hospital and Harvard Medical School, Charlestown, Massachusetts, USA.

**Keywords:** blast overpressure, interface astroglial scarring, special operations forces

## Abstract

Emerging evidence from autopsy studies indicates that interface astroglial scarring (IAS) at the gray–white matter junction is a pathological signature of repeated blast brain injury in military personnel. However, there is currently no *in vivo* neuroimaging test that detects IAS, which is a major barrier to diagnosis, prevention, and treatment. In 27 active-duty U.S. Special Operations Forces personnel with high levels of cumulative blast exposure, we performed translocator protein (TSPO) positron emission tomography (PET) using [^11^C]PBR28 to detect neuroinflammation at the cortical gray–white matter interface, a neuroanatomic location where IAS has been reported in autopsy studies. TSPO signal in individual Operators was compared with the mean TSPO signal in a control group of nine healthy civilian volunteers. We identified five Operators (18.5%) with TSPO signal at the cortical gray–white matter interface that was more than 2 standard deviations above the control mean. Cumulative blast exposure, as measured by the generalized blast exposure value, did not differ between the five Operators with elevated TSPO signal and the 22 Operators without elevated TSPO signal. While the pathophysiologic link between neuroinflammation and IAS remains uncertain, these preliminary observations provide the basis for further investigation into TSPO PET as a potential biomarker of repeated blast brain injury.

## Introduction 

Repeated blast exposure (RBE) is associated with a broad spectrum of neuropathology in United States (U.S.) military personnel.^1,2^ Human autopsy studies^1,3^ and animal models^4,5^ indicate that interface astroglial scarring (IAS) at the gray–white matter junction is a signature lesion of RBE, but there is currently no diagnostic test to detect IAS in military personnel prior to brain autopsy. The absence of an *in vivo* diagnostic test for IAS undermines efforts to prevent and develop therapies for repeated blast brain injury (rBBI).^6^

Translocator protein (TSPO) positron emission tomography (PET) is a potential biomarker for IAS because elevated TSPO can reflect neuroinflammation, which is believed to precede IAS.^7^ Importantly, the pathophysiologic pathways that may link neuroinflammation to IAS have not been fully elucidated,^8^ and the extent to which neuroinflammation contributes to post-traumatic pathologies such as IAS remains uncertain.^7,9^ Moreover, it is well established that reactive astrocytes have heterogeneous functional properties,^10^ depending on the type, duration, and frequency of the challenge to neuronal homeostasis (e.g., single blunt trauma, repetitive blunt trauma, single blast trauma, or RBE).^7^ Accordingly, neuroinflammation may be adaptive, leading to healing and restoration of neuronal function,^11^ or maladaptive, leading to neurodegeneration.^7^ In this context, the clinical implications of neuroinflammation detected by TSPO PET must be interpreted with caution. Nevertheless, in the absence of an *in vivo* diagnostic test for IAS, TSPO PET provides an opportunity for early detection of neuroimmune alterations that may contribute to the pathogenesis of IAS.

Thus, we performed a TSPO PET analysis to identify individual Operators with increased TSPO signal at the cortical gray–white matter interface—a neuroanatomic region predisposed to IAS. Our goal in this proof-of-principle analysis was to determine whether TSPO PET should be further investigated as an early imaging biomarker of IAS, and hence rBBI, in future studies of blast-exposed military personnel.

## Methods

The present work is a secondary analysis of the dataset acquired in the ReBlast Pilot study—Long-Term Effects of Repeated Blast Exposure in U.S. Special Operations Forces (SOF) Personnel.^12^ The ReBlast Pilot study protocol was preregistered at ClinicalTrials.gov (NCT05183087), and a detailed description of the study protocol was previously published.^13^ All participants provided written informed consent, in accordance with a protocol approved by the Mass General Brigham Institutional Review Board and the U.S. Special Operations Command Human Research Protections Office. We enrolled 30 active-duty U.S. SOF who met the following inclusion criteria: (1) male; (2) age 25–45 years; (3) active-duty SOF; (4) prior combat deployment; (5) prior combat exposure during deployment, verified by the Combat Exposure Scale^14^; and (6) exposure to blast overpressure, verified by an interview-based version of the generalized blast exposure value (GBEV).^15^ We excluded individuals with (1) moderate–severe traumatic brain injury and (2) imaging contraindication.

SOF personnel traveled to the Massachusetts General Hospital Athinoula A. Martinos Center for Biomedical Imaging for 2 days, during which [^11^C]PBR28 TSPO PET-magnetic resonance imaging (MRI) was performed as part of a multimodal assessment.^13^ [^11^C]PBR28 TSPO data were acquired on a hybrid PET-MRI scanner, the Siemens BrainPET, which uses a head-only PET camera inserted into the bore of a 3 Tesla TIMTrio MRI scanner.^16^ A T1-weighted multi-echo magnetization-prepared rapid gradient-echo (MEMPRAGE)^17^ scan was acquired at 1 mm isotropic resolution with prospective motion correction^18^ for PET attenuation correction and anatomical localization of the TSPO signal.

[^11^C]PBR28 emission data collected 60–90 min post-radioligand injection were divided into 5 min frames, reconstructed to standardized uptake value (SUV) images using an MRI-based attenuation map, realigned,^19^ and averaged (SUV_60–90_). The SUV_60–90_ image was then linearly registered to the participant’s MEMPRAGE scan using FreeSurfer’s spmregister,^20^ skull-stripped, and normalized by the whole brain without ventricles^20^ to account for individual differences in global signal (SUVR_60–90_). Three participants with a TSPO genotype that confers low-affinity binding for [^11^C]PBR28 were excluded from the TSPO PET analyses, yielding a sample size of 27 for this analysis. Additional details regarding TSPO tracer synthesis, PET-MRI data acquisition and quality assessment, TSPO genotyping, and PET reconstruction have been previously described.^12^

The civilian control group was acquired as part of a separate study protocol^20^ approved by the Mass General Brigham Institutional Review Board, with written informed consent provided by healthy volunteers. Controls had no history of a neurological disorder, head trauma, or serious medical illness. For comparison with the SOF cohort, we identified nine controls who were male and whose age was ≥25 years (i.e., the minimum age in the inclusion criteria for the SOF cohort). All controls were imaged on the same PET-MRI scanner, and all PET-MRI and T1 MEMPRAGE acquisition, processing, and analysis parameters for the control group were identical to those used for the SOF cohort.

To generate a cortical gray–white matter interface region of interest (ROI), we processed the SOF and control MEMPRAGE datasets using FreeSurfer,^21^ created labels for gray and white matter, and then created a new ROI along the entire cortical gray–white matter interface. The outer boundary of the ROI was created by expanding the mesh surface at the gray–white interface by a distance equal to 25% of the cortical thickness. The inner boundary was created by contracting the surface into the adjacent white matter (again by 25% of the cortical thickness). Voxels between these two boundaries were taken as the gray–white matter interface ROI.

To identify individual Operators with significantly elevated TSPO signal within the gray–white matter interface ROI compared with the control group, we identified individuals with a *z*-score above 2 (i.e., 2 standard deviations [SD] above the control mean). We calculated the proportion with elevated TSPO and an exact binomial 95% confidence interval; we used the exact binomial test to assess whether the observed proportion of individual Operators with elevated TSPO was different from the expected proportion of Operators with elevated TSPO, under the null hypothesis that Operators were part of the normal distribution derived from the control dataset (null proportion = 0.023). We also assessed differences in demographics, blast exposure, combat exposure, and blunt head impacts in Operators with and without increased TSPO signal within the cortical gray–white interface ROI. We used Wilcoxon rank sum tests for continuous variables and Fisher’s exact tests for categorical variables.

## Results

Demographic and exposure data for the 27 Operators are provided in the Table. The mean ± SD age of the nine controls was 30.4 ± 4.5 years. With respect to race, six controls were white, two were Asian, and one reported more than one race. In terms of ethnicity, two controls were Hispanic and seven were non-Hispanic.

**FIG. 1.**
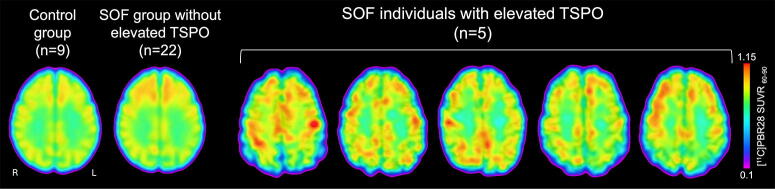
Translocator protein (TSPO) positron emission tomography (PET) detects neuroinflammation at the cortical gray–white matter interface in individual Operators. Axial images of TSPO PET data are shown for the five individual Operators with elevated TSPO signal at the cortical gray–white matter interface, compared with the 22 Operators without elevated TSPO signal and to a control group of nine healthy civilians. For the control group and the 22 Operators without elevated TSPO signal, the mean TSPO PET signal map is shown in MNI standard space. The intensity of the TSPO signal at the cortical gray–white matter interface is indicated by the color bar. L, left; R, right; SUVR_60–90_, standardized uptake value normalized by whole brain mean, based on emission data collected from 60–90 min post-radioligand injection for [^11^C]PBR28.

**FIG. 2.**
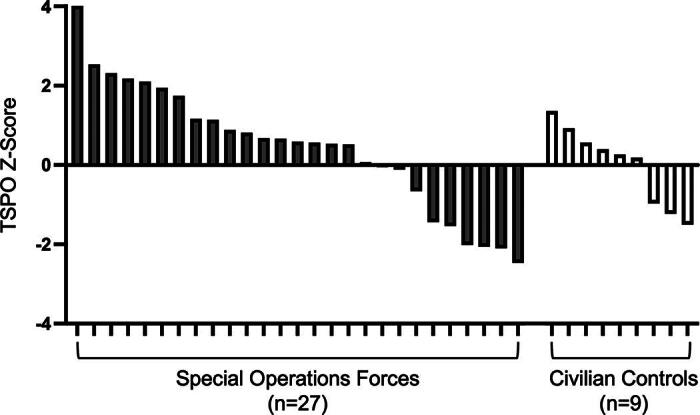
Translocator protein (TSPO) positron emission tomography (PET) *z*-score distributions for special operations forces and civilian controls. All TSPO measurements were performed at the gray–white matter interface of the cerebral cortex. *Z*-Scores represent the number of standard deviations that an individual’s TSPO signal differs from that of the control mean.

Five of 27 Operators had a TSPO signal at the cortical gray–white matter interface that was at least 2 SD above the control mean (Fig. 1). The proportion of Operators with elevated TSPO was 0.185 (5/27), with a 95% confidence interval of [0.063, 0.381], and when we tested whether the observed proportion was different from 0.023, the *p* value was 0.0003. The distribution of *z*-scores for all 27 Operators and nine controls is provided in Figure 2.

The GBEV scores of the five Operators with elevated TSPO values ranged from 389,317 to 38,129,760 with a median value of 19,960,106. Four of five had experienced heavy combat, and one had experienced moderate combat, as measured by the Combat Exposure Scale.^14^ All five had been exposed to explosive blasts within the past year. Four out of five had more blunt contact blows to the head than they could recall, as assessed by the Brain Injury Screening Questionnaire.^22^ There were no significant differences in demographics or exposure to blast, blunt head trauma, or combat between the five Operators with elevated TSPO signal and the 22 without elevated TSPO signal at the cortical gray–white matter interface (Table 1).

**Table 1.** Clinical, Demographic, and Exposure Characteristics

Characteristic	SOF with non-elevated TSPO (*n* = 22)	SOF with elevated TSPO (*n* = 5)	*p*
Age, years	36.5 ± 4.1	39.6 ± 3.4	0.34
Sex: Male, no.	22	5	NA
Race: White, no.	22	5	NA
Ethnicity: non-Hispanic, no.	19	5	1.00
Years in service	16.5 ± 4.8	19.2 ±3.0	0.47
Military branch, no.	15 Army	4 Army	0.66
	4 Navy	0 Navy	
	2 Air force	0 Air force	
	1 Marine	1 Marine	
Rank, no.			1.00
Officer	1	0	
Warrant officer	3	1	
Enlisted	18	4	
CES score	33.7 ± 4.9	35.2 ± 3.4	0.90
Combat exposure (CES), no.			0.14
Moderate	1	1	
Moderate-heavy	9	0	
Heavy	12	4	
Blows to the head (BISQ), no.			0.64
Low	8	1	
High	14	4	
Cumulative blast exposure (GBEV) median (range)	10,041,994 [1,430,877–363,812,869]	19,960,106 [389,317–38,129,760]	0.84
Most recent blast exposure, no.			1.00
<1 year	18	5	
1 year	2	0	
2 years	2	0	

Blows to the head “high” = number of participants with more blows to the head than they could remember; “low” = number of participants who could recall a finite number of blows to the head (range of blows to the head: 1–13), assessed by the Brain Injury Screening Questionnaire (BISQ). Combat exposure was assessed using the Combat Exposure Scale (CES). Continuous variables were statistically compared between the two SOF groups using Wilcoxon rank sum tests for continuous variables and Fisher’s exact tests for categorical variables.

GBEV, generalized blast exposure value; NA, not applicable; SOF, Special Operations Forces; TSPO, translocator protein.

## Discussion

In this proof-of-principle TSPO PET analysis of active-duty U.S. SOF, TSPO signal at the cortical gray–white matter interface was elevated in 5 of 27 Operators (18.5%) compared with a group of healthy civilian controls. There were no significant differences in demographics, blast exposure, blunt head trauma exposure, or combat exposure in Operators with elevated TSPO at the gray–white interface compared with those without elevated TSPO at the gray–white matter interface. While the pathophysiological relationship between neuroinflammation and IAS at the gray–white interface remains uncertain, these observations provide the basis for further investigation of TSPO PET as a potential early biomarker of rBBI.

The presence of increased TSPO signal at the gray–white matter interface in active-duty U.S. SOF should be interpreted in the context of evolving concepts about the mechanistic complexity of TSPO signal.^23^ While TSPO has been classically interpreted as a measure of neuroinflammation because of its expression within reactive microglia and astrocytes, there is growing recognition that TSPO may be interpreted in the context of cellular metabolism, given that it is expressed within the mitochondria of neurons, endothelial cells, and vascular smooth muscle cells.^24^ It is therefore possible that changes in the TSPO signal may require different mechanistic interpretations, depending on the context.

Our interpretation of increased TSPO signal as a sign of neuroinflammation in the present study is consistent with that of a prior study in a different cohort of blast-exposed SOF personnel.^25^ However, in our previously reported group-level analysis of the same 27 individuals analyzed here,^12^ we found that increased blast exposure was associated with decreased TSPO in the rostral anterior cingulate cortex (rACC). In this latter context, we interpreted decreased TSPO signal as reflecting mitochondrial-driven metabolic dysfunction within the rACC, particularly because a corresponding increase in rACC cortical thickness was believed to be a potential manifestation of IAS.

Collectively, these observations indicate that regional alterations in the TSPO signal may reflect distinct pathophysiological processes (i.e., neuroinflammation versus decreased cellular metabolism). It is possible that increased TSPO signal (i.e., neuroinflammation) represents an early, treatable stage of rBBI, whereas decreased TSPO signal (i.e., decreased cellular metabolism) may represent a late stage of rBBI in which IAS has already occurred. While our goal in the present study was to identify Operators with elevated TSPO signal at the cortical gray–white matter interface, we also identified Operators with low signal, highlighting the challenge of interpreting cross-sectional TSPO PET data in active-duty Operators with high levels of blast exposure. Future longitudinal studies of dynamic TSPO signal changes, and their correlation with blast exposure, are needed to clarify the temporal dynamics of these potential pathophysiologic mechanisms.

It is also important to consider that pathophysiologic manifestations of neuroinflammation in the human brain are variable^8,10^ and cannot be determined solely based on an increase in the TSPO signal. There is mechanistic plausibility for a relationship between neuroinflammation and IAS, given the role of reactive, dysfunctional, and/or dysmorphic astrocytes in IAS pathogenesis,^5^ but it is also possible that the TSPO signal observed here represents an adaptive neuroinflammatory response to brain injury. Indeed, when adaptive, neuroinflammation may lead to the healing of injured neurons and restoration of neuronal function after brain injury.^7^ This potential beneficial role of neuroinflammation is highlighted by the results of a recent clinical trial, in which reduction of neuroinflammation by the anti-inflammatory agent minocycline was associated with increased levels of neurodegenerative proteins in the blood in civilians with traumatic brain injury.^26^ Elucidation of whether neuroinflammation is adaptive or maladaptive in blast-exposed military personnel will require multimodal, longitudinal studies with complementary neuroimaging, blood, cognitive, psychological, and pathology endpoints.^27^

The potential link between RBE and neuroinflammation may be influenced by unmeasured risk factors that could confer susceptibility or resilience, or additional exposures. Indeed, SOF personnel experience myriad exposures during training and combat, such as inhalation of heavy metal fumes, acceleration g-forces, and hypoxia,^6^ that were not measured in the present study. Moreover, GBEV scores for the 27 SOF personnel in this study ranged from 389,317 to 38,129,760, which is higher than a previously reported GBEV threshold (200,000) at which military personnel experience cognitive, psychological, or physical symptoms.^15^ It is therefore possible that a statistical association between cumulative blast exposure and TSPO signal may have been masked by the uniformly elevated GBEV scores. A key direction for future studies will be to identify the risk factors and exposures that mediate the potential associations between RBE, neuroinflammation, and IAS.

In summary, we found preliminary evidence of neuroinflammation, as measured by TSPO PET, at the cortical gray–white interface—the same region where IAS has been previously described^1,28^—in five of 27 active-duty U.S. SOF. Given the small sample size, the use of a civilian control cohort, and the unmeasured additional exposures, these results should be considered preliminary and hypothesis-generating. The presence of increased TSPO signal at the gray–white matter interface does not prove a causal link between neuroinflammation and IAS and, in the absence of autopsy data, it is not possible to determine whether any of the Operators in this study had a diagnosis of IAS. Nevertheless, these observations provide the basis for further investigation of TSPO PET as a potential biomarker of neuroinflammation in blast-exposed military personnel. Future studies are needed to clarify which risk factors predispose to neuroinflammation, to determine whether neuroinflammation is a reliable biomarker of rBBI, and to identify predictors of brain healing versus scarring when neuroinflammation is detected in military personnel.

## Abbreviations Used

BISQ: Brain Injury Screening Questionnaire
CES: Combat Exposure Scale
GBEV: Generalized Blast Exposure Value
IAS: interface astroglial scarring
MEMPRAGE: multi-echo magnetization-prepared rapid gradient-echo
MRI: magnetic resonance imaging
PET: positron emission tomography
rACC: rostral anterior cingulate cortex
rBBI: repeated blast brain injury
RBE: repeated blast exposure
ROI: region of interest
SOF: Special Operations Forces
SUV: standardized uptake value
TSPO: translocator protein
U.S.: United States
